# Diagnostic accuracy of tuning fork weber test and audiometric weber test in school-aged children with conductive hearing loss

**DOI:** 10.1007/s00405-026-10325-6

**Published:** 2026-06-10

**Authors:** Tee Shao Peng, Nik Adilah Nik Othman, Mohd Normani Zakaria, Nik Fariza Husna Nik Hassan

**Affiliations:** 1 Department of Otorhinolaryngology – Head and Neck Surgery, School of Medical Sciences, Health Campus Universiti Sains Malaysia, 16150 Kubang Kerian, Kelantan, Malaysia; 2 Department of Otorhinolaryngology – Head and Neck Surgery, Hospital Sultan Ismail, Taman Mount Austin, Jalan Mutiara Emas Utama81100 Johor Bahru, Johor, Malaysia; 3 Audiology Programme, School of Health Sciences, Health Campus Universiti Sains Malaysia, 16150 Kubang Kerian, Kelantan, Malaysia

**Keywords:** Tuning fork Weber, Audiometric Weber, Air–bone gap, Conductive hearing loss

## Abstract

**Purpose:**

While the application of tuning fork Weber (TFW) test in adult is well-documented, its accuracy in the paediatric population remains uncertain although conductive hearing loss is frequently identified in children. Similarly, the effectiveness of the audiometric Weber (AW) test is ambiguous. Given evidence suggesting that age influences lateralization accuracy and understanding of instructions, this research specifically targets school-aged children to evaluate these diagnostic tools effectively. This study aims to explore the lateralization accuracy of TFW and AW tests in detecting conductive hearing loss in children and the agreement of these tests with the predicted lateralization responses based on audiogram.

**Methods:**

This study involved 75 children with conductive hearing loss aged between seven to twelve years old. Each subject underwent TFW (256 Hz and 512 Hz) and AW (250 Hz and 500 Hz) tests.

**Results:**

All subjects achieved high accuracy (> 80%) in both TFW and AW tests. McNemar’s test revealed there was no significant difference between the TFW and AW tests for both frequencies (*p* > 0.05). Using Cohen’s Kappa, the lateralization responses in both tests demonstrate substantial to almost perfect agreement (*k* = 0.78–0.92). with the predicted lateralization responses for both frequencies. Large air–bone gap group also demonstrated higher accuracy for both tests compared to the small air–bone gap group, although the difference was not statistically significant (*p* > 0.05).

**Conclusion:**

Both the TFW and AW tests showed high lateralization accuracy in either unilateral or bilateral CHL supported by substantial to almost perfect agreement with the respective PTA in school-aged children with conductive hearing loss.

## Introduction

Conductive hearing loss (CHL) predominantly occurs in children (90–95%) compared to adults [1]. Among the common causes of CHL in children are otitis media with effusion and Eustachian tube dysfunction. Early ENT intervention and management of CHL in children is imperative, as it can cause delay in speech and language development and cognition which can subsequently affect their school performance and learning capability [2].

Tuning Fork test has been widely used since ancient medical practice. The Tuning Fork Weber (TFW) test is named after Ernst Heinrich Weber, an anatomist and physiologist in Germany [3]. He found out that sound source conducted via skull bone will increase at both ears equally if both ear canals were occluded, but if only one ear canal was occluded, only the occluded ear will increase in sensation. Theoretically, there are two phenomena that will cause lateralization during the Weber test. The first phenomenon is the lateralization to the better cochlear function ear in the event of bilateral sensorineural hearing loss. The second phenomenon will lateralize to the conductive pathology ear provided that ear has a normal cochlear function. In the case of bilateral conductive pathology, the TFW test will lateralize to the side with the greater air–bone gap (ABG) in pure tone audiometry (PTA). The disadvantages of the TFW test are that the bone conduction is affected by the thickness of the cranial bone, the tension of the skin, and the amount of hair on the skin. Besides that, tuning fork that is made of steel will become oxidised and rusty if exposed to moist environment unless well kept in a dry area. Certain tuning fork has nickel plating coated and it will begin to peel off after prolonged usage. All these changing properties of tuning fork will affect the pitch and produce adventitious sound.

The Audiometric Weber (AW) test applies the same concept and principle as the TFW test. The former will be using an audiometer bone vibrator replacing the tuning fork as an investigative tool. The advantage of using an audiometer bone vibrator over a tuning fork is the ability to produce consistent intensity at a desirable level without fading off [4]. Besides that, there is a lesser possibility of the vibration sound being transmitted by air conduction to the subject if compared to TFW test [4].

In a study done on 6 to 15-year-old bilateral NH children using a 256 Hz tuning fork, 98.2% of them were able to identify the tone heard in midline correctly. When tested on children with unilateral CHL, 62.5% of the responses lateralized accurately [5]. In a similar study conducted on children aged 4 to 10 with bilateral otitis media with effusion, 62% responded correctly [6]. In contrast, the accuracy of TFW test was found to be poor in 2 to 11-year-old children with otitis media with effusion. There was no significant association between the ABG and the lateralization of the TFW test [2]. The reasons given are a lack of understanding or judgement to complete the test and inadequate instruction given. A similar result was reported by Yung and Morris. They concluded that tuning fork testing is more effective to perform on children above 4years old [7]. There is sufficient evidence of the association of age with the lateralization accuracy to warrant further investigation.

To date, literature concerning the accuracy of TFW and AW tests among children population is still rather scarce. The use of the AW test is also elusive. Researchers are keen to evaluate the concordance of both the Weber tests with PTA-predicted lateralization responses in detecting CHL, as the time taken to perform the Weber tests is much shorter than completing PTA. In view of the possible limitations of PTA such as masking dilemma and “false” ABG, we would like to find out the Weber tests application in justifying the questionable audiogram.

## Material and methods

This cross-sectional study involved 75 children diagnosed with CHL aged between seven to twelve years old. They were recruited from the Otorhinolaryngology Clinic in Hospital Sultan Ismail and Hospital Universiti Sains Malaysia. Children who have unilateral CHL (with ABG of at least 15 dB at 250 Hz and 500 Hz) or bilateral asymmetrical CHL (with the difference between right and left air conduction thresholds of at least 15 dB at two adjacent frequencies) were included. Those who have wound and skin diseases affecting the tuning fork and bone vibrator placement and were unable to provide appropriate behavioural responses due to conditioning failure were excluded.

Predicted Weber lateralization was determined based on PTA findings. In unilateral cases, lateralization was predicted toward the ear with conductive pathology. In asymmetrical CHL cases, lateralization was expected toward the ear with the larger ABG.

### Procedure

Hearing assessment was carried out to confirm that subjects fulfil the inclusion criteria. Otoscopic examination, PTA, and tympanometry test were carried out by audiologist. Tympanometry was used to confirm the presence of conductive element. The tester was blinded from the PTA result before performing the TFW and AW tests. The tester then obtained the consent from the parent or guardian before proceeding with the TFW and AW tests.

**Fig. 1:**
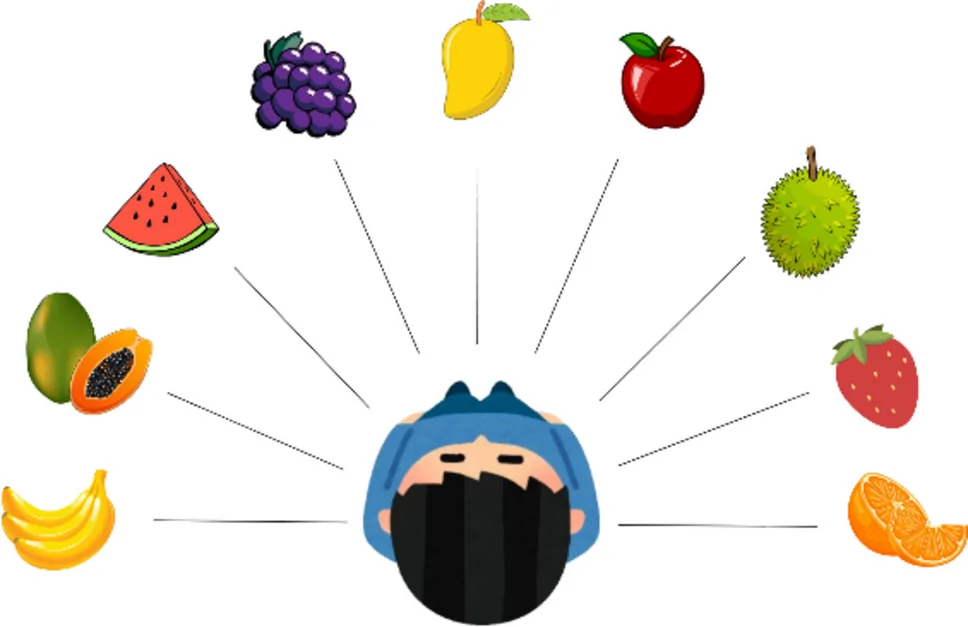
Visual aid

In order to get reliable results, all subjects were subjected to a conditioning process before the Weber test was performed to make sure they understand what would be conducted later. The tester played a stimulus with left or right lateralization through a headphone. The subjects were guided to point out the lateralization on the visual aid (Fig. 1) which consisted of nine markers. The lateralization was taken as “left” if the subject pointed at the four leftmost markers whereas taken as “right” if the subject pointed at the four rightmost markers. If the subject chose the middle marker which was represented by a mango, it was classified as “central”.

This visual aid was developed and tested prior to this study. Fruit images were selected because they are age-appropriate and easily recognizable, thereby minimizing confusion related to participants’ prior knowledge. However, it has not undergone psychometric validation and should therefore be interpreted with caution.

TFW and AW tests using different frequencies were randomised in order to avoid the order effect. All tests were also completed during the same visit to prevent time bias.

### TFW test

The TFW test was carried out in a quiet room with an ambient noise level lower than 60 dB SPL. The tuning fork was struck at the distal one-third of the prong at the tester’s olecranon. Then, the vibrating footplate of the tuning fork was placed firmly at the vertex of the skull vault. The task of the subject was to determine whether the tone heard was perceived on the left, right, or midline of the head by either pointing or verbally. Responses for 256 Hz and 512 Hz were recorded.

### AW test

Subject were seated upright and the bone vibrator B71 was placed at the midline of the patient’s forehead. The tester presented the bone vibrator with a 500 Hz pure tone at 20 dB SL (intensity 20 dB above the threshold of that particular frequency) using the audiometer. The subjects were instructed to identify whether the tone was perceived in the left, right, or central position. The procedure was repeated using 250 Hz pure tone.

### Statistical analysis

All the data collected were analysed using SPSS version 25. McNemar’s test was used to compare the paired proportions between the TFW and AW tests at equivalent frequencies (256 Hz vs 250 Hz and 512 Hz vs 500 Hz), whereas Cohen’s Kappa coefficient was used to evaluate the agreement between the lateralization responses (TFW and AW tests) and the predicted lateralization responses. Descriptive analysis was used to classify the ABG into small or large ABG group.

## Result

75 children with hearing loss (47 males and 28 females) with the age ranging from 7 to 12 years old (mean age of 8.83 ± 1.68 years) were enrolled in this study. 57 subjects have unilateral CHL (76%), and 18 subjects have bilateral asymmetrical CHL (24%).

The overall accuracy of correct lateralization for the TFW test was 96% and 92% for 256 Hz and 512 Hz respectively. On the other hand, the accuracy for AW test was 93.33% and 97.33% for 250 Hz and 500 Hz respectively. All subjects achieved high accuracy (> 80%) in both TFW and AW tests as shown in Table 1. Statistical comparison using McNemar’s test showed no significant difference between the TFW and AW tests at both frequencies (*p* > 0.05).

**Table 1:** Accuracy and Kappa agreement results for TFW and AW tests

	TFW test			AW test			
	Frequency (Hz)	Accuracy (%)	Kappa	Frequency (Hz)	Accuracy (%)	Kappa	*p*-value
Overall	256	96.00	0.92	250	93.33	0.78	0.687
	512	92.00	0.78	500	97.33	0.90	0.219
Unilateral CHL	256	96.50	0.93	250	94.70	0.81	1.000
	512	91.20	0.76	500	96.50	0.87	0.375
Bilateral CHL	256	94.40	0.88	250	88.90	0.66	1.000
	512	94.40	0.87	500	100	1.00	-

As for the agreement with the 250 Hz PTA, the kappa values were 0.92 for 256 Hz TFW and 0.78 for 250 Hz AW. Comparatively, the kappa values were 0.78 for 512 Hz TFW and 0.90 for 500 Hz AW with respect to the agreement with 500 Hz PTA. The lateralization responses between the TFW and AW tests showed substantial to almost perfect agreement with the predicted lateralization responses at both frequencies.

To examine the significance of ABG in the lateralization of Weber in TFW and AW tests in CHL children, a median value was identified to classify the subjects into large and small ABG groups. The median values for 250 Hz and 500 Hz were 45 dB and 40 dB respectively. Those with ABG in the worse ear lower than the median ABG value were grouped into the small ABG group, and vice versa.

Referring to 250 Hz on the audiogram for the worse ear, 37 and 38 participants were classified into small and large ABG groups respectively, whereas for 500 Hz, there were 27 and 48 participants grouped into small and large ABG groups respectively.

Comparisons between the ABG groups showed no significant result for both TFW and AW tests in both frequencies (*p* > 0.05). However, the large ABG group was found to provide higher accuracy for both tests as shown in Table 2. It was also noted that TFW test had higher accuracy at 256 Hz and AW test had higher accuracy at 500 Hz. Confidence interval upper bounds slightly exceeding 1.0 were observed due to asymptotic approximation methods and were not truncated, as the estimates are not strictly bounded at 1.

**Table 2:** Comparison of accuracy and agreement using Cohen’s Kappa coefficient between small and large ABG groups

	TFW test				AW test				
	Frequency (Hz)	Accuracy (%)	Kappa	95% CI	Frequency (Hz)	Accuracy (%)	Kappa	95% CI	*p*-value
Overall	256	96.00	0.92	0.85–1.00	250	93.33	0.78	0.61–0.95	0.687
	512	92.00	0.78	0.62–0.94	500	97.33	0.90	0.76–1.03	0.219
Small ABG	256	94.59	0.90	0.78–1.02	250	91.89	0.71	0.45–0.98	1.000
	512	88.89	0.75	0.52–0.99	500	96.30	0.86	0.61–1.11	0.500
Large ABG	256	97.37	0.95	0.85–1.05	250	94.74	0.85	0.65–1.05	1.000
	512	93.75	0.80	0.59–1.00	500	97.92	0.92	0.76–1.07	0.625

## Discussion

The present study was conducted to assess the concordance of the AW and TFW tests with PTA-predicted lateralization responses. The findings demonstrated that both the TFW (256 Hz and 512 Hz) and AW (250 Hz and 500 Hz) tests showed satisfactory accuracy (> 88%) in identifying unilateral or bilateral asymmetrical CHL in children aged 7 to 12 years. This was further demonstrated by Kappa values, which indicated substantial to almost perfect agreement at 250 Hz and 500 Hz with the respective PTA results. These findings contrast with the previous reports suggesting poor reliability of TFW tests in children. [2, 6].

Behn et al. reported poor accuracy of the TFW test in a cohort of 58 children with OME aged 2 to 11 years (mean age: 5.8 years). Similarly, Capper et al. found a correct response rate of 62% when performing the 512 Hz TFW test in children with a mean age group of 5 to 7 years with bilateral OME. In contrast, the present study focused on older children aged 7 to 12 years. This group was more cooperative and better able to understand the task. This difference in age range may explain the higher reliability observed in our findings.

Thompson et al. evaluated the accuracy of the AW test in the CHL group and reported an accuracy of 76.5% for unilateral CHL and 40% for bilateral CHL. However, their study differed methodologically from ours. Their sample included a broader age range (12 to 75 years), and only the 1000 Hz was tested [8]. These differences may account for the variation in findings.

Our results are consistent with those reported by Abdullah et al., who studied individuals aged 12 to 67 years. They demonstrated an overall accuracy exceeding 80% for both TFW and AW tests across frequencies, which is comparable to our findings [9]. This suggests that school-aged children are capable of performing these tasks at a level similar to adults.

Litovsky et al. reported that children typically attain adult-like sound localization abilities between the ages of four and five [10]. This may partly explain the high accuracy observed in the present study. In addition, the use of a conditioning process likely contributed to improved performance. The visual aid used in this study helped subjects indicate sound lateralization more accurately. It reduced confusion between left, right, and central responses and minimized errors. However, as this visual aid is newly developed, further validation is required to establish its reliability, validity, and clinical applicability. Another contributing factor may be the use of a single experienced tester, which ensured consistent test administration.

All tests in this study were conducted using standardized steel tuning forks. Steel tuning forks provide better bone conduction efficiency compared to aluminium forks due to superior mechanical vibration energy output [11]. In contrast, aluminium tuning forks are more susceptible to metal fatigue after repeated use. This may alter their acoustic properties, including fundamental frequency, overtones, and decay time [12].

Furthermore, the present study found no significant difference in accuracy between TFW and AW tests across frequencies and ABG groups. However, some differences in agreement were observed. TFW at 256 Hz (k = 0.92) showed higher agreement compared to AW at 250 Hz (*k* = 0.78). Conversely, AW at 500 Hz (k = 0.90) demonstrated better agreement than TFW at 512 Hz (*k* = 0.78). These findings suggest that TFW at 256 Hz and AW at 500 Hz may provide more reliable lateralization results. Nevertheless, these observations should be interpreted with caution, as the underlying causes remain unclear. Further studies with larger sample sizes and diverse hearing loss profiles are needed.

The observed discrepancies may be related to technical factors associated with the instruments. The bone vibrator B71 is known to produce harmonic distortion at low frequencies, particularly at 250 Hz [13, 14]. Similarly, tuning forks exhibit complex vibroacoustic behaviour. For example, the stem may oscillate at an octave frequency, which is double the fundamental frequency, even though the prongs do not. Non-linear harmonic frequencies may also occur when the fork is struck against a hard surface, resulting in large vibration amplitudes. These effects can be minimized by striking the tuning fork on a softer surface [15].

Consistent with previous studies, the larger ABG group demonstrated higher accuracy compared to the small ABG group for both TFW and AW tests [6, 16]. This may be due to a more pronounced conductive component, which enhances the lateralization effect. Previous studies have shown that Weber lateralization can occur with an ABG as small as 2.5 to 4 dB [5, 6]. Therefore, all subjects in this study were expected to demonstrate correct lateralization. However, several factors may have influenced accuracy. These include inconsistent responses from children, undetected concurrent sensorineural hearing loss, and limitations of the instruments used.

The limitation in the present study was that both the Weber tests was restricted to low frequencies (250 Hz and 500 Hz). Further investigation in higher frequency would be valuable. Low-frequency testing is particularly sensitive to variations in the static application force of the bone vibrator. Small changes in force can result in significant intensity differences at 250 Hz, compared to higher frequencies such as 2000 Hz [18].

This finding revealed the probable pitfall of the bone vibrator and tuning fork in delivering constant pressure force at the forehead. Variations in placement may lead to changes in perceived loudness, particularly at low frequencies. Khanna et al. found that a small shift of the vibrator can significantly affect sound pressure level below 2000Hz. They also reported lower sensation levels by 10–15 dB when the bone vibrator is placed on the forehead compared to the mastoid[19].

Dirk recommended that a force of at least 400 g should be applied when placing the bone vibrator on the forehead. However, this was not measured in the present study [20]. Additionally, the vibrotactile sensitivity and harmonic distortion present in the bone vibrator B71 might affect accuracy at the lower frequencies, potentially leading to overestimation of ABG [13, 14]. Future study should explore the newer bone vibrator B81 which offers improved electroacoustic properties.

Specific aetiologies of conductive hearing loss were also not analysed in this study, which may influence the ABG magnitude. The study also did not include a normal-hearing control group or participants with sensorineural or mixed hearing loss. Furthermore, the visual aid used has not undergone formal validation, and inter-rater variability was not assessed. These factors may limit the interpretability of the findings and should be considered in future studies.

## Conclusion

Both the TFW and AW tests showed high accuracy in either unilateral or bilateral CHL supported by substantial to almost perfect agreement with the respective PTA despite the test being conducted in school-going children. While supporting its established role in ENT practice as a reliable clinical adjunct, it is crucial to emphasize that the Weber test should not replace a formal PTA. It should serve as a confirmation test in the event of masking dilemma or suspicious ABG as well as be supplemented by other objective or subjective audiological investigations on the lookout for a definitive diagnosis.

## Data Availability

The datasets used and/or analysed during the current study are available from the corresponding author on reasonable request.
